# Black Aorta from Alkaptonuria

**DOI:** 10.1055/s-0041-1729916

**Published:** 2021-10-29

**Authors:** James P. Laurent, Sean D. Galvin

**Affiliations:** 1 Department of Cardiothoracic Surgery, Wellington Regional Hospital, Wellington, New Zealand

**Keywords:** aorta, alkaptonuria, ochronosis, aortic valve replacement

## Abstract

A-76-year old male with a past history of alkaptonuria with ochronosis (homogentisic acid deposition in tissues) had symptomatic aortic stenosis. Surgical replacement of the valve was undertaken, and he was noted to have a severely pigmented and porcelain aorta.

A 76-year-old gentleman was referred for aortic valve surgery due to severe aortic stenosis with New York Heart Association class II symptoms of dyspnea and exercise intolerance. Past medical history included alkaptonuria with ochronosis ( Figs. 1 and 2 ), severe osteoarthritis requiring bilateral hip, knee, and shoulder replacements, diabetes mellitus, hypercholesterolemia, and mild renal dysfunction. An echocardiogram showed severe aortic stenosis with a mean gradient of 47 mm Hg and an area of 0.6 cm ^2^ . Coronary angiography showed moderate disease in the diagonal and first obtuse marginal arteries.

**Fig. 1:**
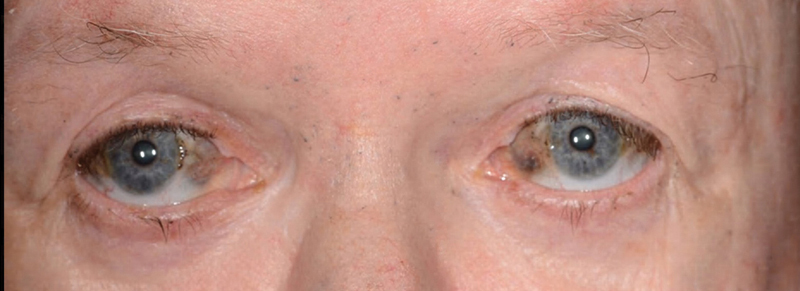
Ochronosis deposition of sclera.

**Fig. 2:**
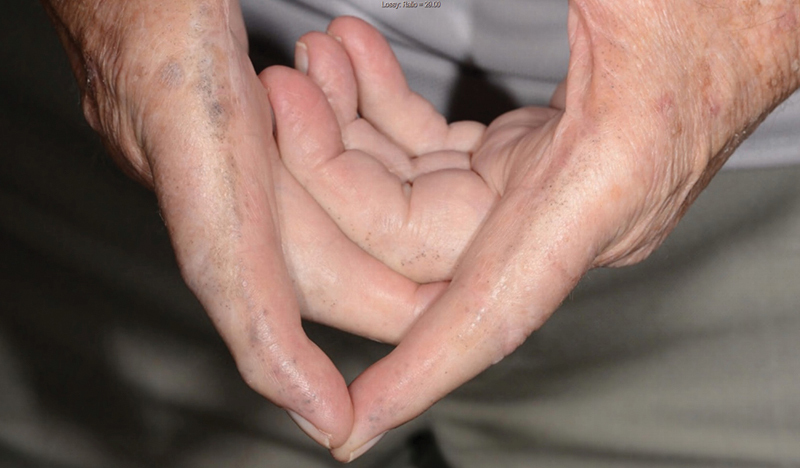
Ochronosis deposition of the fingers.

He proceeded to an elective surgical aortic valve replacement. On performing the sternotomy, the xiphisternum, cartilage, and costochondral joints were noted to be black ( Fig. 3 ). The sternal tissues were very fragile and friable. The aorta along with the entire arterial tree was dark blue to black in color. On palpation of the aorta. it was noted to have significant plaque that was confirmed on epiaortic ultrasonography. This was predominately over the anterior wall of the aorta extending up into the arch and down into the root.

**Fig. 3:**
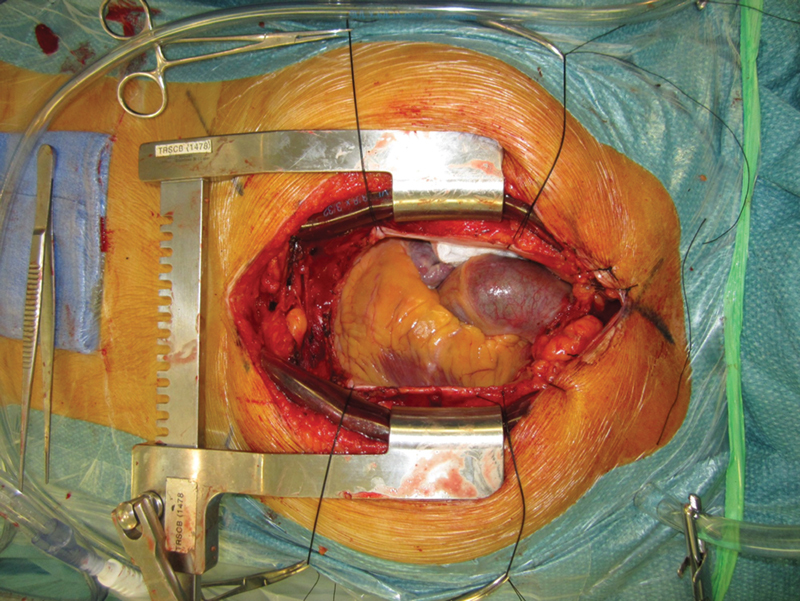
Intraoperatvie findings of the pigmented aorta.

It was felt unsafe to proceed with clamping of the aorta, and the procedure was abandoned. He was subsequently referred for a transfemoral aortic valve implant with a good result.

There have been no complications with this gentleman for the last 24 months.

Alkaptonuria is an autosomal-recessive metabolic disorder due to a defect in the homogentisic acid dioxygenase enzyme, leading to widespread deposition of polymeric homogentisic acid. Pigment deposition is the hallmark of the disease, with the intima and adventitia of the aorta being primarily involved and the media relatively spared. This case report is unique in showing the adventitia of the aortic wall being heavily pigmented. Prior case reports have only shown pigmented valves and intima of the aorta.

